# The Prevalence and Indications of Intravenous Rehydration Therapy in Hospital Settings: A Systematic Review

**DOI:** 10.3390/epidemiologia4010002

**Published:** 2022-12-31

**Authors:** Julia Gawronska, Ai Koyanagi, Guillermo F. López Sánchez, Nicola Veronese, Petre Cristian Ilie, Anne Carrie, Lee Smith, Pinar Soysal

**Affiliations:** 1 The Cambridge Centre for Sport and Exercise Science, Anglia Ruskin University, Cambridge CB1 1PT, UK; 2 Research and Development Unit, Parc Sanitari Sant Joan de Déu, CIBERSAM, ISCIII, Dr. Antoni Pujadas, 08830 Sant Boi de Llobregat, Spain; 3 ICREA, Pg. Lluis Companys 23, 08010 Barcelona, Spain; 4 Division of Preventive Medicine and Public Health, Department of Public Health Sciences, School of Medicine, University of Murcia, 30120 Murcia, Spain; 5 Geriatrics Section, Department of Internal Medicine, University of Palermo, 90133 Palermo, Italy; 6 Research and Innovation Department, The Queen Elizabeth Hospital Foundation Trust, King’s Lynn PE30 4ET, UK; 7 Centre for Health Performance and Wellbeing, Anglia Ruskin University, Cambridge CB1 1PT, UK; 8 Department of Geriatric Medicine, Faculty of Medicine, Bezmialem Vakif University, 34093 Istanbul, Turkey

**Keywords:** intravenous fluids therapy, IV rehydration, paediatric patients, adult patients

## Abstract

(1) Objective: We performed a systematic review to explore the prevalence of intravenous (IV) rehydration therapy in hospital settings, and we assessed it by patient groups and populations. (2) Methods: A systematic review of major databases and grey literature was undertaken from inception to 28 March 2022. Studies reporting prevalence of IV rehydration therapy in a hospital setting were identified. The data were synthesised in a narrative approach. (3) Results: Overall, 29 papers met the inclusion criteria. The prevalence of IV rehydration therapy in paediatric patients ranged from 4.5% (hospitalised with diarrhoea and dehydration) to 100% (admitted to the emergency department with mild to moderate dehydration caused by viral gastroenteritis), and in adults this ranged from 1.5% (had single substance ingestion of modafinil) to 100% (hospitalised with hypercalcemia). The most common indication for IV rehydration therapy in paediatric patients was dehydration due to fluid loss from the gastrointestinal tract. Other causes included malnutrition, neuromuscular disease, bronchiolitis, and influenza. In adults, indications for IV rehydration therapy were much more diverse: fever, diarrhoea, drug intoxication, hypercalcemia, cancer, and postural tachycardia syndrome; (4) Conclusions: This systematic review showed that IV rehydration therapy in paediatric patients is often used to treat dehydration and diarrhoea, while in adults it has a broader spectrum of use. While IV rehydration therapy is important in correcting fluid problems and electrolyte status, the maintenance fluid prescribing practices vary considerably, and guidelines are scarce.

## 1. Introduction

Intravenous (IV) rehydration therapy is widely used to prevent or correct problems with fluid and electrolyte status when oral administration is not possible, or it is impaired [1]. IV fluids enter the bloodstream directly, bypassing the waiting time associated with oral rehydration. Fluid loss can be caused by surgery, accident, or common conditions such as fever, vomiting, and diarrhoea. Moreover, in some cases requiring hospitalisation such as acute renal failure, hyponatremia, hypercalcemia, acute pancreatitis, and sepsis, which are more common in adults, IV rehydration is the most important part of the treatment and can be lifesaving, even if there is no loss of fluid [2,3]. In addition, IV hydration is used to maintain hydration in paediatric and adult cancer patients or terminal care patients who do not have adequate oral intake [4]. Therefore, IV rehydration therapy has a wide range of uses.

IV rehydration therapy is the procedure by which a specially formulated IV solution is administrated through a tube attached to a needle, which is inserted into a vein. IV solutions contain small amounts of salt (sodium chloride) or sugar (dextrose, glucose, or levulose) that are dissolved in sterile water [1]. One of the most used IV fluids is 0.9% normal saline that contains sterile water and 0.9% sodium chloride. IV rehydration therapy is a simple and effective way of supplying fluids directly into the intravascular fluid compartment. However, an interprofessional team approach is sometimes required to achieve optimum fluid balance for patients [5]. The type, amount, and infusion rate of IV rehydration therapy may vary according to body composition, dehydration level, and cardiac output status of each patient, as well as clinical and hemodynamic parameters such as daily urine output or blood pressure. Therefore, fluid prescription may be difficult, especially in patients with impaired homeostatic mechanisms, such as those with renal or heart failure, or in patients with ongoing excessive losses (e.g., as a result of diarrhoea) [6]. Moreover, incorrect management of fluid assessment and monitoring is associated with adverse outcomes such as hyponatremia (sodium concentration of less than 135 mmol/L (135 mEq/L)), fluid overload, and hyperchloremic acidosis (pH less than 7.35 develops with an increase in ionic chloride) due to inappropriate fluid composition and/or infusion rates/volumes [2]. For example, despite the obvious benefits of IV fluid therapy, excessive fluid administration may lead to various complications [7]. High volumes of IV fluids may be retained in the interstitial space, causing interstitial oedema, impaired organ perfusion, possibly acute pulmonary oedema, and increased mortality [7]. Optimal fluid status not only shortens hospital stay, but may also reduce the incidence of postoperative complications, mortality, and adverse outcomes related to dehydration, such as acute confusion, constipation, urinary tract infections, fatigue, falls, and delayed wound healing, particularly in older adults [8]. Dehydration has also been associated with longer hospital stays, with an annual cost estimate of >1.14 billion USD in 1999 for the diagnosis of primary dehydration [9]. All this clearly shows how important interventions to improve hydration are in older adults.

However, to date, no attempt has been made to collate the literature on the prevalence of IV rehydration therapy in hospital settings, and little is known about this, particularly by patient groups or populations. Information on prevalence of IV rehydration therapy in hospital settings is of upmost importance to aid in hospital decision making (procurement and medical practice), the development of policy, and medical guidelines (i.e., is it over or under used). Therefore, in this systematic review, we provide an overview of the prevalence of IV rehydration therapy in hospital settings in paediatric and adult populations.

## 2. Materials and Methods

The current systematic review followed the Preferred Reporting Items for Systematic Reviews and Meta-Analyses (PRISMA) guidelines [10]. Prior to conducting our review, we identified the following questions to guide our search:

The following two questions were established to guide the search:

1. What is the prevalence of IV rehydration therapy in hospital settings?
2. Is there a certain age group of people that is more likely to receive IV rehydration therapy in hospital settings?

### 2.1. Search Strategy

Electronic databases were searched from database inception until 28 March 2022 including PubMed/Medline, Embase, Web of Science, and Scopus. In PubMed, the following search strategy was used: “(“intravenous fluids”[Title/Abstract] OR “parenteral fluids”[Title/Abstract] OR “IV fluids”[Title/Abstract] OR “fluid infusion”[Title/Abstract] OR “fluid administration”[Title/Abstract] OR “fluid therapy”[Title/Abstract] OR “fluid perfusion”[Title/Abstract] OR “intravenous rehydration”[Title/Abstract] OR “parenteral rehydration”[Title/Abstract]) AND (“hospitalised patients”[Title/Abstract] OR “hospitalisation”[Title/Abstract] OR “in hospital”[Title/Abstract])”. The strategy was then adapted for the other databases. Full information on database-specific search strategies can be found in online Supplemental Table S1. Results of the searches were exported to bibliographic database and duplicates removed. Titles and abstracts were screened by two independent reviewers (JG, LS), and then the full paper screening was conducted by the same reviewers before making a final decision on eligibility. Any inconsistencies were discussed and resolved by consensus with a third reviewer (PS).

### 2.2. Study Inclusion and Exclusion

Studies were included if they met the following criteria: (1) observational cross-sectional, prospective, or retrospective cohort studies (2) that investigate the frequency of IV rehydration therapy (3) in any population (healthy or with a specific disease condition) (4) in a hospital setting. Published articles that were written in English were included. Review articles, nonhuman studies, conference abstracts, and articles in a language other than English were excluded from the review. Studies investigating IV therapy for resuscitation and studies that reported on IV rehydration solutions with added medications into the IV bolus and goal-directed fluid therapy were excluded.

Studies were excluded at full text for the following criteria: (1) IV fluids for resuscitation, (2) fluid overload related to IV fluids, (3) hyponatraemia related to IV fluids, (4) guided IV therapy or restricted IV fluid therapy, (5) intraoperative/postoperative IV fluid administration, (6) hydration by enteroclysis, (7) outpatient IV rehydration, (8) IV fluids use at home, (9) use of IV fluids not specified, (10) RCT, or (11) combined data for IV fluids and oral rehydration solution.

### 2.3. Data Extraction

Data were extracted by an independent reviewer (JG) including the following: first author, year, country, type of the study design (cross-sectional, cohort), population, sample size included, participants’ characteristics (e.g., age, sex), number of participants receiving IV rehydration therapy, information of control/comparator group, and the period of observation. A second independent reviewer (LS) validated the data extraction. The data were synthesised in a narrative approach.

### 2.4. Quality Assessment

Risk of bias in individual studies was assessed by one independent reviewer (JG) and checked by another (LS) using the Critical Appraisal Skills Programme [11]. The Cohort Study checklist was used for the cohort studies [12]. This checklist contains 12 questions to which the reviewer answered ‘yes’, ‘cannot tell’, or ‘no’. The Cohort Study checklist was also used for the cross-sectional studies because there is no separate cross-sectional survey checklist in the CASP series. Questions 6(a) and 6(b), which assessed the follow up of the study, were not applicable to the cross-sectional designs and were therefore marked as not applicable. The quality was evaluated as ‘fair’, ‘good’, and ‘poor’ based on the CASP checklists. Any discrepancies were resolved during a discussion with a third reviewer (PS).

## 3. Results

### 3.1. Search Results

**Figure 1:**
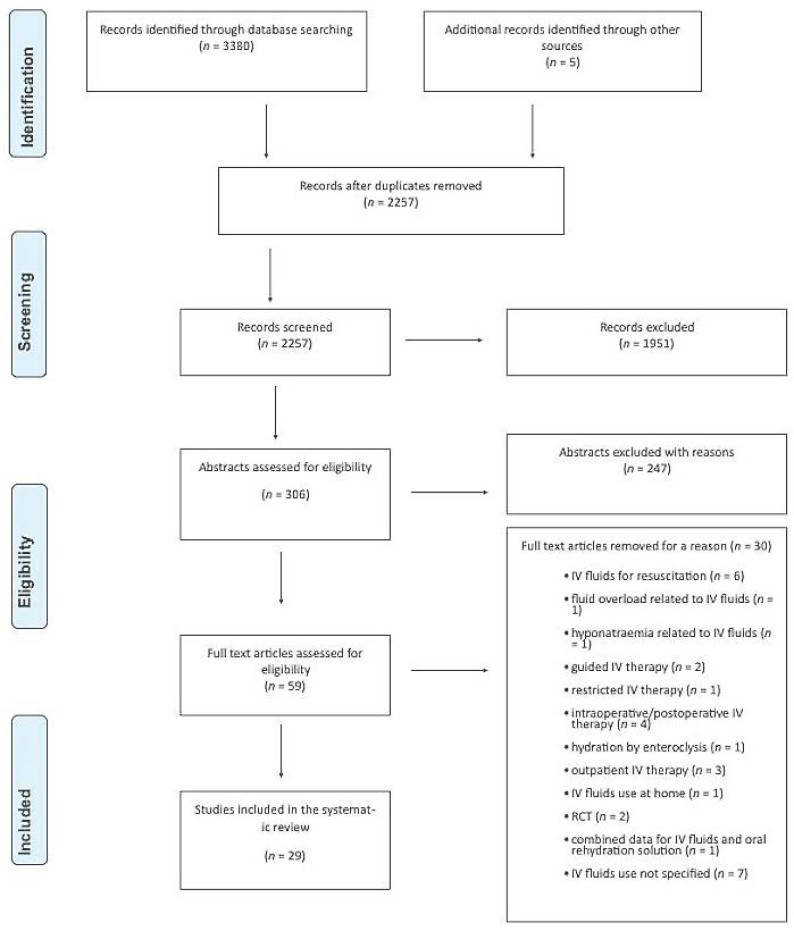
PRISMA flow diagram illustrating article selection.

Of 2257 articles screened, we reviewed the full text of 59 studies. After careful review, the authors agreed on the inclusion of 29 studies for the narrative synthesis. Figure 1 shows the PRISMA flow diagram.

### 3.2. Studies’ Characteristics

All included studies were published between 1991 and 2021. The 29 included studies yielded a total of 863,346 patients and the age ranged from 3 days to 87 years old. Study characteristics can be found in Table 1. A total of 51.5% were male.

Regarding populations, 21 studies were conducted on paediatric patients. Four studies reported the prevalence of IV rehydration in children admitted to the hospital with diagnosis of diarrhoea [13,14,15,16]; ten studies considered children admitted to the hospital with diagnosis of acute gastroenteritis [17,18,19,20,21,22,23,24,25,26]; three studies considered children hospitalised with dehydration [27,28,29]; one study considered children admitted to the hospital with chronic neuromuscular disorder [30]; one study considered children admitted to the hospital with severe acute malnutrition [31]; one study considered children hospitalised with laboratory-confirmed influenza [32]; and one study considered children hospitalised with bronchiolitis [33].

Eight studies were conducted on adult patients. One study investigated the prevalence of IV rehydration in patients with incurable cancer [34]; two studies investigated patients with fever and other signs of dengue [35,36]; one study investigated patients admitted to the cholera isolation ward [37]; one study investigated patients who had single substance ingestion of modafinil [38]; one study investigated patients with diarrhoea [39]; one study investigated patients with hypercalcemia [40]; and one study investigated patients with postural tachycardia syndrome [41].

**Table 1:** Demographic characteristics of included studies.

Author, Year	Country or Region	Study Duration	Study Design	Sample Size	Age Range	Age Mean (SD)	Age Median (IQR)	Sex % Male	Population	Quality
Abdul-Mumin, Ervin and Halvorson, 2019 [18]	Ghana	January 2013–December 2014	Retrospective chart review	473	NR	NR	12 (9–24) months	56	Paediatric patients hospitalised with acute gastroenteritis	Good
Akech et al., 2018 [13]	Kenya	October 2013–December 2016	Prospective chart review	8025	NR	NR	12 (8–18) months	0	Paediatric patients hospitalised with diarrhoea and dehydration	Good
Ben- Shalom, Toker and Schwartz, 2016 [29]	Israel	2001–2010	Retrospective chart review	58	0–24 months	6.8 (5.27) months	NR	59.7	Paediatric patients hospitalised with hypernatremic dehydration	Good
Blacklock et al., 2015 [38]	Sierra Leone	26 July 2012–22 September 2012	Retrospective chart review	798	<5–≥60 years	NR	NR	45	Paediatric and adult patients hospitalised with cholera during the epidemic	Fair
Chow et al., 2009 [14]	China	1 April 2021 to 31 March 2003	Retrospective chart review	7391	NR	NR	13 (6–26) months	59	Paediatric patients admitted to the hospital with diagnosis of diarrhoea.	Fair
Dbaibo et al., 2013 [19]	Lebanon	April 2007–August 2008	Hospital-based surveillance design	491	NR	NR	12 (0–59) months	NR	Paediatric patients hospitalised with a diagnosis of acute gastroenteritis	Fair
Fikrie, Alemayehu and Gebremedhin, 2019 [32]	Ethiopia	July 2015–June 2017	Retrospective cohort	381	6–59 months	22.4 (15.8)	NR	49.6	Paediatric patients hospitalised with Severe Acute Malnutrition	Good
Freedman et al., 2014 [20]	USA	1 January 2002–31 December 2011	Retrospective cohort study	804,000	NR	3.1 (3.9) years	NR	53.1	Paediatric patients who were diagnosed as having gastroenteritis in an emergency department	Good
Heyman et al., 1990 [15]	Malawi	July 1981–July 1986	Retrospective chart review	3495	≤12–≥24 months	NR	NR	77	Paediatric patients hospitalised with diarrhoea or gastroenteritis	Fair
Janet el al., 2015 [30]	Spain	15 July 2012–15 December 2012	Prospective cohort study	83	NR	NR	4 (1.7–7) years	56.6	Paediatric patients with mild-to moderate isonatremic dehydration	Fair
Kao et al., 2019 [31]	Taiwan	January 2005–January 2015	Retrospective chart review	44	NR	9.9 (5.6) years	11.1 (10.6)	68.2	Paediatric patients with chronic neuromuscular disorder who visited the emergency room	Good
Lopez-Medina et al., 2012 [33]	USA	27 April 2009–23 March 2010	Retrospective cohort study	73	3–179 days	NR	48 days	48	Paediatric patients hospitalised with laboratory-confirmed influenza	Fair
Machado et al., 2015 [41]	USA	1 October 2010–30 September 2013	Retrospective chart review	72	54–87 years	70.4 (NR) years	NR	40	Patients hospitalised with hypercalcemia	Good
Marra et al., 2011 [36]	Brazil	8 April 2008–9 May 2008	Retrospective chart review	3393	NR	NR	NR	NR	Paediatric and adult patients treated in the hydration tent during dengue fever epidemic	Fair
Moineau and Newman, 1990 [21]	Canada	December 1988–April 1989	Prospective pilot study	17	NR	2.6 (1.7) years	NR	47	Paediatric patients admitted to the emergency department with mild to moderate dehydration caused by viral gastroenteritis	Fair
Myat et al., 2021 [22]	Myanmar	May 2018–January 2020	Hospital-based surveillance design	3226	5 days to 59 months	NR	NR	59.5	Paediatric patients hospitalised for acute gastroenteritis	Fair
Nazurdinov et al., 2018 [23]	Tajikistan	January 2013–December 2014	Hospital-based surveillance design	2863	0–59 months	NR	NR	61	Paediatric patients hospitalised with acute gastroenteritis and rotavirus	Fair
Oakley et al., 2016 [34]	Australia	1 April to 31 October 2011 to 2013	Retrospective cohort study	491	NR	5.1 (1.9) weeks	NR	56	Paediatric patients hospitalised with bronchiolitis	Fair
Patwari et al., 1991 [16]	India	January 1989–December 1989	Retrospective chart review	5996	0–5 years	NR	NR	64.9	Paediatric patients who attended hospital with diarrhoea	Fair
Perl et al., 2011 [24]	Israel	1 April 2004–31 March 2006	Retrospective chart review	533	NR	21.7 (31) months	13 months	56.5	Paediatric patients hospitalised with acute gastroenteritis, rotavirus gastroenteritis, and diarrhoea and vomiting	Good
Redondo-Gonzalez et al., 2016 [25]	Spain	1 January 2003–31 December 2009	Retrospective cohort study	17,415	7 months–≥14 years	NR	NR	53.4	Paediatric patients hospitalised with acute gastroenteritis	Fair
Spiller et al., 2009 [39]	USA	2000–2007	Retrospective chart review	137	1–82 years	22 (NR) years	NR	38	Adult patients who had single substance ingestion of modafanil	Fair
Hasan et al., 2021 [40]	Bangladesh	2 April 2018–12 May 2018	Retrospective chart review	1531	0–≥ 30 years	NR	NR	58.4	Paediatric and adult patients hospitalised with diarrhoea during epidemic	Good
Tewari et al., 2018 [37]	New Delhi	May 2013–September 2013	Prospective cohort study	500	6 months to 77 years	NR	NR	53.9	Paediatric and adult patients hospitalised with fever and other signs of dengue	Fair
Thronaes et al., 2021 [35]	Norway	15 January 2019–15 January 2020	Prospective longitudinal study	451	NR	68.9 (13.1) years	NR	60.3	Adult patients with incurable cancer	Fair
Tseng et al., 2018 [42]	USA	January 2010–January 2017	Retrospective cohort study	332	NR	29.3 (9.5) years	NR	10	Adult patients’ postural tachycardia syndrome	Fair
Waisbourd-Zinman et al., 2008 [26]	Israel	1 January 2003–31 December 2006	Prospective cohort study	356	NR	14.6 (24.7) months	9 months	54.5	Paediatric patients hospitalised with nosocomial rotavirus gastroenteritis	Fair
Wathen, MacKenzie and Bothner, 2004 [28]	USA	January–October, 2004	Prospective cohort study	182	2.7 months to 8.5 years		1.4 years	51	Paediatric patients presenting at the hospital with gastroenteritis and dehydration	Fair
Wildi-Runge et al., 2009 [27]	Switzerland	July 2002–March 2006	Retrospective chart review	539	NR	1.4 (NR) years	NR	55.6	Paediatric patients hospitalised with rotavirus gastroenteritis	Fair

SD, standard deviation; IQR, interquartile range; NR, not reported.

### 3.3. Frequency of Intravenous Rehydration in Paediatric Patients

Of the 29 included studies, 21 reported the prevalence of IV rehydration in paediatric patients. This review found that the use of IV rehydration therapy varied considerably amongst paediatric patients. The prevalence ranged from 4.5% to 100% (Table 2).

Four studies reported the prevalence of IV rehydration in children admitted to the hospital with diagnosis of diarrhoea [13,14,15,16]. The prevalence ranged from 6.1% to 48%. Five studies reported the prevalence of IV rehydration in children admitted to the hospital with diagnosis of acute gastroenteritis [17,19,20,25,27]. The prevalence ranged from 18.5% to 100%. Six studies reported the prevalence of IV rehydration in children with diagnosis of acute gastroenteritis who also tested positive for rotavirus [18,21,22,23,24,26]. The prevalence in children with rotavirus ranged from 4.5% to 100%. Two studies reported the prevalence of IV rehydration in children hospitalised with dehydration [28,29]. The prevalence was 100%. One study reported the prevalence of IV rehydration in children admitted to the hospital with chronic neuromuscular disorder [30]. The prevalence was 34%. One study reported the prevalence of IV rehydration in children admitted to the hospital with severe acute malnutrition [31]. The prevalence was 22.8%. One study reported the prevalence of IV rehydration in children hospitalised with laboratory-confirmed influenza [32]. The prevalence was 53%. One study reported the prevalence of IV rehydration in children hospitalised with bronchiolitis [33]. The prevalence was 31%.

These results show that the highest prevalence of IV rehydration therapy was observed amongst those hospitalised with dehydration [28,29] and rotavirus-positive gastroenteritis [18], while the lowest prevalence was also observed amongst those hospitalized with acute gastroenteritis but only amongst rotavirus-negative patients [24]. This suggests that type and severity of illness may play a role in the prevalence of IV rehydration therapy. Furthermore, patients with rotavirus-positive gastroenteritis were administrated IV rehydration therapy more often compared to rotavirus-negative patients.

This review showed that the most common indication for IV rehydration therapy in paediatric patients was dehydration due to fluid loss from the gastrointestinal tract. The second most common indication for IV rehydration was influenza, followed by neuromuscular disease, bronchiolitis, and malnutrition. 

### 3.4. Frequency of Intravenous Rehydration in Adult Patients

Of the 29 included studies, 8 reported the prevalence of IV rehydration in adult patients. Similar to the paediatric patients, the use of IV rehydration therapy varied considerably amongst adult patients. The prevalence ranged from 1.5% to 100% (Table 2).

Two studies [35,36] reported the prevalence of IV rehydration in patients with fever and other signs of dengue. The prevalence ranged from 9.2% to 24.3%. One study [39] reported the prevalence of IV rehydration in patients admitted to the hospital with diarrhoea. The prevalence was 51%. One study [34] reported the prevalence of IV rehydration in patients with incurable cancer. The prevalence was 45%. One study [38] reported the prevalence of IV rehydration in patients who had single substance ingestion of modafinil. The prevalence was 1.5%. One study [37] reported the prevalence of IV rehydration in patients admitted to the cholera isolation ward. The prevalence was 96.1%. One study [40] reported the prevalence of IV rehydration in patients hospitalised with calcium supplement syndrome. The prevalence was 22%. One study [41] reported the prevalence of IV rehydration in patients hospitalised with postural tachycardia syndrome. The prevalence was 6.3%.

The results show that the highest prevalence of IV rehydration therapy was observed amongst patients hospitalised with cholera [37], whilst the lowest prevalence of IV rehydration therapy was seen amongst patients who had single substance ingestion of modafanil [38]. We also found a relatively high prevalence of IV rehydration amongst patients with diarrhoea [39]. Although, this review showed that IV rehydration was required in all paediatric patients suffering from dehydration [20,27], IV therapy amongst dehydrated adult patients was not so frequent [35].

This review indicated that in adult patients, the most common indication for IV rehydration therapy was dehydration due to fluid loss caused by cholera, and the second most common indication for IV rehydration was dehydration caused by diarrhoea, followed by cancer, fever, hypercalcemia, postural tachycardia syndrome, and drug intoxication.

## 4. Discussion

This systematic review of 29 studies demonstrated that there are substantial differences in the prevalence of IV rehydration therapy in both paediatric and adult populations. The prevalence of IV rehydration therapy in paediatric patients ranged from 4.5% to 100% and in adult patients ranged from 1.5% to 100%. In paediatric patients, IV rehydration therapy was required more frequently (80%) due to dehydration owing to fluid loss from the gastrointestinal tract, while other causes included malnutrition, neuromuscular disease, bronchiolitis, and influenza. In adults, indications for IV rehydration therapy were much more diverse: fever, diarrhoea, drug intoxication, hypercalcemia, cancer, and postural tachycardia syndrome.

Acute gastroenteritis is a disease with high morbidity and mortality affecting the paediatric population. Dehydration is the most common complication of acute gastroenteritis and therefore a frequent reason for consultation in paediatric emergency departments [29]. The most appropriate treatment method is still in debate. One of the main discussions is regarding the volume and the rate of administration of fluid used for IV rehydration, leading to great variability in the practice in paediatric emergency care [2]. We found that the highest prevalence of IV rehydration therapy was observed amongst paediatric patients who were hospitalised with dehydration [28,29] and rotavirus gastroenteritis [18]. Another finding is that in general, it appears that IV rehydration is much more frequently needed in rotavirus-positive than rotavirus-negative patients. Compared to patients with rotavirus-negative gastroenteritis, patients with rotavirus-positive gastroenteritis had a higher incidence of vomiting, lethargy, and dehydration. However, interestingly, the lowest prevalence of IV rehydration therapy was also seen amongst those hospitalised with rotavirus gastroenteritis [24]. This may be because a wide age range of children (7 months–14 years) were included in this study. On the other hand, dehydration may develop not only in enteritis, but also in the course of other infections in paediatric patients and IV rehydration may be needed. For example, in infants infected with influenza, both nutrition and fluid intake decrease due to fever and respiratory abnormalities, and nausea and diarrhoea increase fluid loss. Moreover, bacterial and viral co-infections can exacerbate these conditions. Therefore, half of hospitalised infants undergo IV rehydration [32]. Although nasogastric hydration has been found to be safe and effective in infants hospitalised for bronchiolitis, it is known that 31% of them are administered IV fluid hydration, and these are sicker infants who are followed up in the intensive care unit and receive IV antibiotic therapy [33]. One of the most important problems in children with neuromuscular diseases or in children in underdeveloped countries (based on malnutrition) is weakness/fatigue, infections, and metabolic disturbances, and all of these can deteriorate the general health status of patients and cause dehydration [30,31]. Therefore, dehydration requiring IV rehydration treatment is present in 23% of patients with severe acute malnutrition and 34% of children with neuromuscular disease admitted to the emergency department [30,31]. Considering the aforementioned data, it becomes clear that there are many factors, such as severity of illness and concomitant infections, affecting IV rehydration therapy in paediatric practice, and treatment should be individualised.

Regarding the adult population, the highest prevalence of IV rehydration therapy was observed amongst patients hospitalised with cholera, and almost all patients admitted received IV fluids, because there was a history of vomiting, which may have influenced the decision to treat with IV fluids in these patients; thus, they could not consume oral rehydration solution [37]. Whilst the lowest prevalence (1.5%) of IV rehydration therapy was seen amongst patients who had single substance ingestion of modafanil [38]. Additionally, patients with postural tachycardia syndrome often have gastrointestinal symptoms, and sometimes, these symptoms can be so severe that non-oral nutritional/hydration support, including IV fluids, may be required. Tseng et al. found this ratio as 6.3% [41]. In the Machado et al.’s study, IV rehydration was administered in all patients positive for calcium supplement syndrome (22% of hypercalcemia cases); supplements were discontinued, and calcium level at discharge was found as normal in 80% of patients [40]. IV rehydration therapies are also frequently used for electrolyte disturbances. However, this review showed that IV rehydration was required mostly for dehydration-related hypernatremia in children, while it was also necessary for hypercalcemia in adults. Although IV rehydration therapies have many clear indications, there are still some dilemmas and ethical issues regarding its role in end of life or palliative care [42]. Withholding and withdrawing hydration from terminally ill patients poses many ethical challenges. For example, from the perspective of Islam, rules governing the care of terminally ill patients are derived from the principle that injury and harm should be prevented or avoided. The hastening of death by the withdrawal of food and drink is forbidden, but Islamic law permits the withdrawal of futile, death-delaying treatment, including life support [43]. In the present review, Thronaes et al. et al., found that IV rehydration was applied in half of incurable cancer patients [34]. Therefore, withholding and withdrawing artificial nutrition and hydration must be evaluated in specific situations (terminally ill patients, palliative care, dementia, aged patients) and always case by case in an individual manner. It is important to treat patients appropriately to their cultural and spiritual needs.

Despite the fact that standard rehydration guidelines for a range of conditions and different settings exist, IV fluids tend to be overutilised. In fact, IV fluids are so ubiquitous in hospitals that one would forget considering the indications. For instance, the World Health Organisation advises the use of oral rehydration to treat mild or moderate dehydration secondary to diarrhoea, and IV rehydration to only treat severe dehydration [44]; many emergency departments and primary care physicians prefer to use IV fluids over oral rehydration solutions for children who are dehydrated [45,46]. Such findings indicate a large gap between clinical guidelines and current clinical practice. Furthermore, Abdul-Mumin [17] reported that 70% of paediatric patients who had no dehydration status at the time of admission received IV fluids. This could reflect both incorrect use of IV solutions and poor evaluation/documentation of hydration status. Therefore, the initial assessment of the patient should also include the decision on whether the patient actually requires IV rehydration, and if that is the case, tailor it to the specific needs of that patient.

The findings of our study should be interpreted within its limitations. First, we had to include highly heterogeneous populations. Moreover, owing to many original studies included in the review not reporting IV rehydration prevalence by definite age-range groups, it was not possible to provide accurate data for prevalence by age group. Future studies reporting prevalence of IV rehydration should attempt to report data by definite age-range groups. Second, evaluation was made independent of the type, content, and infusion rate of IV rehydration therapy. Third, the prevalence and indications of IV rehydration therapy were assessed, but outcomes could not be evaluated. Nonetheless, our systematic review is the first and included a large number of studies regarding important issue on IV rehydration therapy. Finally, while studies reported the condition for which the populations were hospitalised, the exact reasons for hospitalisation (e.g., complications or exacerbations) were not reported.

## 5. Conclusions

In conclusion, IV rehydration therapy can be applied both as a part of the primary treatment in cases caused by dehydration and as a supportive treatment in some conditions, such as drug intoxication, cancer, and postural tachycardia syndrome. While IV rehydration therapy is often implemented owing to dehydration and diarrhoea in children, it is used in a broader spectrum in adults. While IV rehydration therapy is critical, especially for hospitalised patients, maintenance fluid prescribing practices vary considerably, and guidelines are scarce. Therefore, in some cases, an interprofessional team approach is required to achieve optimum fluid balance for these patients.

## Figures and Tables

**Table 2:** Frequency of IV rehydration in paediatric and adult patients.

Author, Year	Sample Size	Exposure Group *n* (%)	Comparator Group *n* (%)	Overall *n* (%)	Effect Size	Population
Chow et al., 2009 [14]	7391	3548 (48%)	-	-	-	Paediatric patients admitted to the hospital with diagnosis of diarrhoea
Patwari et al., 1991 [16]	5996	366 (6.1%)	-	-	-	Paediatric patients who came to the hospital with diarrhoea
Akech et al., 2018 [13]	8025	3569 (45%)	-	-	-	Paediatric patients hospitalised with diarrhoea and dehydration
Heyman et al., 1990 [15]	3495	1310 (37.5%)	-	-	-	Paediatric patients hospitalised with diarrhoea or gastroenteritis
Abdul-Mumin, Ervin and Halvorson, 2019 [17]	473	365 (77%)	-	-	-	Paediatric patients hospitalised with acute gastroenteritis
Freedman et al., 2014 [19]	804,000	148,780 (18.5%)	-	-	-	Paediatric patients who were diagnosed as having gastroenteritis in an emergency department
Moineau and Newman, 1990 [20]	17	17 (100%)	-	-	-	Paediatric patients admitted to the emergency department with mild to moderate dehydration caused by viral gastroenteritis
Wathen, MacKenzie and Bothner, 2004 [27]	182	182 (100%)	-	-	-	Paediatric patients presenting at the hospital with gastroenteritis and dehydration
Waisbourd-Zinman et al., 2008 [25]	356	239 (67%)	-	-	-	Paediatric patients hospitalised with nosocomial rotavirus gastroenteritis
Perl et al., 2011 [23]	533	Rotavirus positive (*n* = 202) 187 (92%)	Rotavirus negative (*n* = 331) 249 (75%)	436 (82%)	4.06 (2.28–7.21)	Paediatric patients hospitalised with acute gastroenteritis, rotavirus gastroenteritis, and diarrhoea, and vomiting
Dbaibo et al., 2013 [18]	491	Rotavirus positive (*n* = 136) 136 (100%)	Rotavirus negative (*n* = 351) 351 (96.3%)	491 (97.4%)	*p* = 0.0234	Paediatric patients hospitalised with a diagnosis of acute gastroenteritis
Nazurdinov et al., 2018 [22]	2863	Rotavirus positive (*n* = 1207) 1097 (91%)	Rotavirus negative (*n* = 1656) 1433 (87%)	2530 (88.5%)	NR	Paediatric patients hospitalised with acute gastroenteritis and rotavirus.
Myat et al., 2021 [21]	2977	Rotavirus positive (*n* = 1320) 770 (58.3%)	Rotavirus negative (*n* = 1657) 880 (53.1%)	1650 (55.5%)	<0.01	Paediatric patients hospitalised for acute gastroenteritis
Redondo-Gonzalez et al., 2016 [24]	17,415	Rotavirus positive (*n* = 1657) 75 (4.5%)	Rotavirus negative (*n* = 15,758) 230 (1.6%)	NR	3.2 (2.46–4.18)	Paediatric patients hospitalised with acute gastroenteritis
Wildi-Runge, 2009 [26]	539	378 (70.1%)	-	-	-	Paediatric patients hospitalised with rotavirus gastroenteritis
Ben- Shalom et al., 2016 [28]	58	58 (100%)	-	-	-	Paediatric patients hospitalised with hypernatremic dehydration
Janet et al., 2015 [29]	83	83 (100%)	-	-	-	Paediatric patients with mild-to moderate isonatremic dehydration
Lopez-Medina et al., 2012 [32]	73	39 (53%)	-	-	-	Paediatric patients hospitalised with laboratory-confirmed influenza
Kao et al., 2019 [30]	44	69 (34%)	**-**	-	-	Paediatric patients with chronic neuromuscular disorder who visited the emergency room
Fikrie, Alemayehu and Gebremedhin, 2019 [31]	381	87 (22.8%)	-	-	-	Paediatric patients hospitalised with Severe Acute Malnutrition
Oakley et al., 2016 [33]	491	65 (31%)	-	-	-	Paediatric patients hospitalised with bronchiolitis
Hasan et al., 2021 [39]	1531	Patients during 2018 epidemic (*n* = 562) 333 (59.3%)	Patients during the seasonally matched periods (*n* = 969) 450 (46.4%)	783 (51%)	OR 95%CI 1.7 (1.4–2.1)	Paediatric and adult patients hospitalised with diarrhoea during epidemic
Marra et al., 2011 [35]	3393	824 (24.3%)	-	-	-	Paediatric and adult patients treated in the hydration tent during dengue fever epidemic
Tewari et al., 2018 [36]	500	45 (9.2%)	-	-	-	Paediatric and adult patients hospitalised with fever and other signs of dengue
Thronaes et al., 2021 [34]	451	203 (45%)	-	-	-	Adult patients with incurable cancer
Spiller et al., 2009 [38]	137	2 (1.5%)	-	-	-	Adult patients who had single substance ingestion of modafanil
Blacklock et al., 2015 [37]	798	767 (96.1%)	-	-	-	Paediatric and adult patients hospitalised with cholera during the epidemic
Machado et al., 2015 [40]	72	Calcium supplement syndrome positive (*n* = 15) 15 (100%)	Calcium supplement syndrome negative (*n* = 57) 0 (0%)	15 (22%)	NR	Patients hospitalised with hypercalcemia
Tseng et al., 2018 [41]	332	21 (6.3%)	-	-	-	Adult patients with postural tachycardia syndrome

*n*, participant; NR, not reported.

## Data Availability

Not applicable.

## Supplementary Materials

The following supporting information can be downloaded at: https://www.mdpi.com/article/10.3390/epidemiologia4010002/s1, Table S1: Database search strategy; Table S2: Risk of bias in cohort studies; Table S3: Risk of bias in cross sectional studies.
