# Supplementary material for: The Prevalence and Indications of Intravenous Rehydration Therapy in Hospital Settings: A Systematic Review
Source: Epidemiologia (Basel). 2022 Dec 31;4(1):18–32. doi: 10.3390/epidemiologia4010002 (PMC9844368; doi:10.3390/epidemiologia4010002)
Supplement: Supplementary file 1 [file epidemiologia-04-00002-s001.zip › epidemiologia-2086940-supplementary.pdf]

Supplementary Table S1. Database search strategy.

**Database**	**Search terms**
Pubmed	("intravenous fluids"[Title/Abstract] OR "parenteral fluids"[Title/Abstract] OR "IV fluids"[Title/Abstract] OR "fluid infusion"[Title/Abstract] OR "fluid administration"[Title/Abstract] OR "fluid therapy"[Title/Abstract] OR "fluid perfusion"[Title/Abstract] OR "intravenous rehydration"[Title/Abstract] OR "parenteral rehydration"[Title/Abstract]) AND ("hospitalised patients"[Title/Abstract] OR "hospitalisation"[Title/Abstract] OR "in hospital"[Title/Abstract])
Embase (searched via OVID)	Title and abstract: ((intravenous fluids or parenteral fluids or IV fluids or fluid infusion or fluid administration or fluid therapy or fluid perfusion or intravenous rehydration or parenteral rehydration) and (hospitalised patients or hospitalisation or in hospital))
Web of Science	"intravenous fluids" OR "parenteral fluids" OR "IV fluids" OR "fluid infusion" OR "fluid administration" OR "fluid therapy" OR "fluid perfusion" OR "intravenous rehydration" OR "parenteral rehydration" AND "hospitalised patients" OR "hospitalisation" OR "in hospital"
Scopus	Title, abstract and keyword: ("intravenous fluids" OR "parenteral fluids" OR "IV fluids" OR "fluid infusion" OR "fluid administration" OR "fluid therapy" OR "fluid perfusion" OR "intravenous rehydration" OR "parenteral rehydration") AND ABS ("hospitalised patients" OR "hospitalisation" OR "in hospital")

Supplementary Table S2. Risk of bias in cohort studies

Author	Q1	Q2	Q3	Q4	Q5a	Q5b	Q6a	Q6b	Q7	Q8	Q9	Q10	Q11	Q12	Overall
Fikrie, Alemayehu and Gebremedhin, 2019	Y	Y	Y	Y	Y	Y	Y	Y	Y	N	Y	N	Y	Y	Good
Freedman et al., 2014	Y	Y	Y	Y	Y	Y	Y	Y	Y	Y	Y	N	Y	Y	Good
Janet el al., 2015	Y	Y	Y	Y	N	N	Y	Y	Y	N	Y	N	Y	Y	Fair
Lopez-Medina et al., 2012	Y	Y	Y	Y	N	N	Y	Y	Y	N	Y	N	Y	Y	Fair
Moineau and Newman, 1990	Y	Y	Y	Y	N	N	Y	Y	Y	N	Y	N	Y	Y	Fair
Oakley et al., 2016	Y	Y	Y	Y	N	N	Y	N	Y	N	Y	N	Y	Y	Fair
Redondo-Gonzalez et al., 2016	Y	Y	Y	Y	Y	Y	Y	N	Y	Y	Y	N	Y	Y	Fair
Tewari et al., 2018	Y	Y	Y	Y	N	N	Y	Y	Y	N	Y	N	Y	Y	Fair
Thronaes et al., 2021	Y	Y	Y	Y	N	N	Y	Y	Y	N	Y	N	Y	Y	Fair
Tseng et al., 2018	Y	Y	Y	Y	N	N	Y	Y	Y	N	Y	N	Y	Y	Fair

Waisbourd-Zinman et al., 2008	Y	Y	Y	Y	N	N	Y	Y	Y	N	Y	N	Y	Y	Fair
Wathen, MacKenzie and Bothner, 2004	Y	Y	Y	Y	N	N	Y	N	Y	N	Y	N	Y	Y	Fair

Q1: Did the study address a clearly focused issue? Q2: Was the cohort recruited in an acceptable way? Q3: Was the exposure accurately measured to minimise bias? Q4: Was the outcome accurately measured to minimise bias? Q5a: Have the authors identified all important confounding factors? Q5b: Have they taken account of the confounding factors in the design and/or analysis? Q6a: Was the follow up of subjects complete enough? Q6b: Was the follow up of subjects long enough? Q7: What are the results of this study? Q8: How precise are the results? Q9: Do you believe the results? Q10: Can the results be applied to the local population? Q11: Do the results of this study fit with other available evidence? Q12: What are the implications of this study for practice? Y, yes; N, no; CT, cannot tell; NA, not applicable.

Supplementary Table S3. Risk of bias in cross sectional studies

Author	Q1	Q2	Q3	Q4	Q5a	Q5b	Q6a	Q6b	Q7	Q8	Q9	Q10	Q11	Q12	Overall
Abdul-Mumin, Ervin and Halvorson, 2019	Y	Y	Y	Y	Y	Y	NA	NA	Y	N	Y	N	Y	Y	Good
Akech et al., 2018	Y	Y	Y	Y	Y	Y	NA	NA	Y	N	Y	N	Y	Y	Good
Ben- Shalom, Toker and Schwartz, 2016	Y	Y	Y	Y	Y	Y	NA	NA	Y	N	Y	N	Y	Y	Good
Blacklock et al., 2015	Y	Y	Y	Y	N	N	NA	NA	Y	N	Y	N	Y	Y	Fair
Chow et al., 2009	Y	Y	Y	Y	N	N	NA	NA	Y	N	Y	N	Y	Y	Fair
Dbaibo et al., 2013	Y	Y	Y	Y	N	N	NA	NA	Y	Y	Y	N	Y	Y	Fair
Heyman et al., 1990	Y	Y	Y	Y	N	N	NA	NA	Y	N	Y	N	Y	Y	Fair
Kao et al., 2019	Y	Y	Y	Y	Y	Y	NA	NA	Y	N	Y	N	Y	Y	Good
Machado et al., 2015	Y	Y	Y	Y	Y	Y	NA	NA	Y	N	Y	N	Y	Y	Good

Marra et al., 2011	Y	Y	Y	Y	N	N	NA	NA	Y	N	Y	N	Y	Y	Fair
Myat et al., 2021	Y	Y	Y	Y	N	N	NA	NA	Y	Y	Y	N	Y	Y	Fair
Nazurdinov et al., 2018	Y	Y	Y	Y	N	N	NA	NA	Y	N	Y	N	Y	Y	Fair
Patwari et al., 1991	Y	Y	Y	Y	N	N	NA	NA	Y	N	Y	N	Y	Y	Fair
Perl et al., 2011	Y	Y	Y	Y	Y	Y	NA	NA	Y	Y	Y	N	Y	Y	Good
Spiller et al., 2009	Y	Y	Y	Y	N	N	NA	NA	Y	Y	Y	N	Y	Y	Fair
Tafsir Hasan et al., 2021	Y	Y	Y	Y	Y	Y	NA	NA	Y	Y	Y	N	Y	Y	Good
Wildi-Runge et al., 2009	Y	Y	Y	Y	N	N	NA	NA	Y	N	Y	N	Y	Y	Fair

Q1: Did the study address a clearly focused issue? Q2: Was the cohort recruited in an acceptable way? Q3: Was the exposure accurately measured to minimise bias? Q4: Was the outcome accu-rately measured to minimise bias? Q5a: Have the authors identified all important confounding factors? Q5b: Have they taken account of the confounding factors in the design and/or analysis? Q6a: Was the follow up of subjects complete enough? Q6b: Was the follow up of subjects long enough? Q7: What are the results of this study? Q8: How precise are the results? Q9: Do you believe the results? Q10: Can the results be applied to the local population? Q11: Do the results of this study fit with other available evidence? Q12: What are the implications of this study for practice? Y, yes; N, no; CT, cannot tell; NA, not applicable.
